# PROMOTING USER TRUST IN AI: THE CASE OF LANGUAGE TECHNOLOGIES

**DOI:** 10.1093/geroni/igae098.0912

**Published:** 2024-12-31

**Authors:** Catherine Diaz-Asper, Chelsea Chandler, Brita Elvevåg

**Affiliations:** Marymount University, Arlington, Virginia, United States; University of Colorado Boulder, Boulder, Colorado, United States; University of Tromsø, the Arctic University of Norway, Tromsø, Nordland, Norway

## Abstract

Advancements in artificial intelligence technology make it possible for computers to recognize, analyze and generate human language in ways that were not possible even five years ago. Approaches such as automated speech analysis, natural language processing, acoustic analysis and machine learning have successfully been applied in various contexts, from clinical research studies predicting cognitive decline in older adults, to chatbots that simulate conversations with users from voice or text input. As speech and language can be easily and rapidly accessed at relatively low cost, these methods hold significant potential for large scale use in commercial, medical and research applications. However, several ethical challenges arise with the use of natural language and speech processing technologies, including issues of transparency, equity and bias, potentially eroding user trust in such tools. For example, language expression varies as a function of demographic factors such as age, culture and education, yet many applications based on these technologies fail to take this into account. The result is that groups who are underrepresented in the data used to train machine learning models are at risk of being unfairly and inaccurately assessed by them. Mistrust can be further exacerbated in groups with reduced knowledge and familiarity with artificial intelligence, as may be the case with older adults and/or those with cognitive complaints. In highlighting both the optimism and challenges of using language technologies with older adults, we present several approaches to encourage confidence in their use, including stakeholder co-design, user guides and graphical interfaces.

